# Cryo-tomography tilt-series alignment with consideration of the beam-induced sample motion

**DOI:** 10.1016/j.jsb.2018.02.001

**Published:** 2018-06

**Authors:** Jose-Jesus Fernandez, Sam Li, Tanmay A.M. Bharat, David A. Agard

**Affiliations:** a Spanish National Research Council (CNB-CSIC), Darwin 3, 28049 Madrid, Spain; b Dept. Biochemistry and Biophysics, University of California, San Francisco, USA; c MRC Laboratory of Molecular Biology, Francis Crick Avenue Cambridge CB2 0QH, UK; d Sir William Dunn School of Pathology, University of Oxford, Oxford OX1 3RE, UK

**Keywords:** Electron cryo-tomography, Beam-induced sample motion, Tilt-series alignment, Tomographic reconstruction, Subtomogram averaging

## Abstract

Recent evidence suggests that the beam-induced motion of the sample during tilt-series acquisition is a major resolution-limiting factor in electron cryo-tomography (cryoET). It causes suboptimal tilt-series alignment and thus deterioration of the reconstruction quality. Here we present a novel approach to tilt-series alignment and tomographic reconstruction that considers the beam-induced sample motion through the tilt-series. It extends the standard fiducial-based alignment approach in cryoET by introducing quadratic polynomials to model the sample motion. The model can be used during reconstruction to yield a motion-compensated tomogram. We evaluated our method on various datasets with different sample sizes. The results demonstrate that our method could be a useful tool to improve the quality of tomograms and the resolution in cryoET.

## 1 Introduction

Recent advancements have established single particle electron cryo-microscopy (cryoEM) as a high-resolution structure determination technique (Kuhlbrandt, 2014, Bai et al., 2015, Vinothkumar and Henderson, 2016, Crowther, 2016). Electron cryo-tomography (cryoET), where multiple cryoEM images of the sample are acquired at different tilts to produce a three dimensional (3D) volume of the field of view, has also experienced a qualitative leap as a structural tool thanks to these innovations along with others specific to the technique (Lucic et al., 2013, Beck and Baumeister, 2016, Wan and Briggs, 2016).

The advent of direct detection detectors (DDDs) has been essential to overcome one of the major resolution-limiting factors in cryoEM: the motion and deformation undergone by the sample during imaging, which produces image blurring and degradation of high-resolution information (Henderson and Glaeser, 1985, Brilot et al., 2012). The electron irradiation induces the doming of the sample, which is caused by a drum-like motion of the ice layer, with substantial translation along the direction perpendicular to the specimen plane (Wright et al., 2006, Brilot et al., 2012, Zheng et al., 2017). This sample deformation produces complex patterns of motion that varies smoothly across the illuminated area and become observable through projection onto the image plane (Brilot et al., 2012, Zheng et al., 2017). The high frame rate of DDDs allows this beam-induced sample motion to be captured in movies. Image processing can be used to track the sample movement throughout the movie frames, align the frames to correct for this movement and sum them up to produce motion-corrected images with high-resolution information restored (Brilot et al., 2012, Campbell et al., 2012, Li et al., 2013, Ripstein and Rubinstein, 2016, Zheng et al., 2017).

Several approaches have been introduced for computational correction of the beam-induced sample motion, which have proved to be instrumental for near-atomic structure determination by single particle cryoEM (Bai et al., 2015). They include correction for the global, uniform movement affecting the whole frame (Li et al., 2013, Grant and Grigorieff, 2015, McLeod et al., 2017) and for the local, smoothly varying, motion (Scheres, 2014, Rubinstein and Brubaker, 2015, Abrishami et al., 2015) (reviewed by Ripstein and Rubinstein (2016)). Recently, a patch-based approach has been introduced that corrects for the anisotropic local motion based on the sample doming model, and further improves the resolution in single particle cryoEM (Zheng et al., 2017).

In cryoET, sample motion is typically corrected for by processing each image in the tilt-series individually as in single particle cryoEM (Wan and Briggs, 2016). Thus, the same motion correction methods are applied to the movies (typically consisting of 3–7 frames) acquired for each tilt image. An important difference is that the SNR of cryoET frames is much lower than in single particle cryoEM as the dose is fractionated over the tilt-series (typically 40–120 images) and the sample thickness increases at high tilts. Under those SNR conditions, whole-frame motion correction methods are commonly used for cryoET, though potentially the recently developed patch-based approach could also be used if enough signal is preserved (Zheng et al., 2017).

Despite the recent significant progress, and apart from a number of exceptional cases (Schur et al., 2016), cryoET is still far behind single particle cryoEM in terms of resolution (Bharat et al., 2015). Several limiting factors remain to be solved (Wan and Briggs, 2016). One of them is sample motion and deformation, added to the fact that the images of the tilt-series represent different fields of view (Bharat et al., 2015). The current workflow in standard cryoET compensates for the sample deformation within each tilt in the tilt-series by correcting for the projection of motion observed at the image plane, but still ignores the deformation that the sample may undergo through the different tilts. This ignored deformation will translate into suboptimal tilt-series alignment and hence deterioration of the quality of the tomogram and its high-resolution information (Voortman et al., 2014, Bharat et al., 2015). Determination and correction for the local sample motion by means of single-particle angular refinement techniques may compensate the tilt-series misalignment and increase the resolution (Bartesaghi et al., 2012, Zhang and Ren, 2012). However, this approach is only applicable to samples where target subtomograms can be tracked through the tilt-series without overlapping with neighbouring features.

In this work, we introduce a method for tilt-series alignment that considers the beam-induced sample deformation through the tilt-series. It relies upon fiducial markers to estimate deformation, which is modelled by polynomial surfaces that represent motions in different directions. Subsequent tomographic reconstruction then takes into account this deformation. We evaluate the new method by using several datasets with different sample sizes. We demonstrate that the new method is able to improve the accuracy of the tilt-series alignment, the quality of tomograms and the resolution of the subtomogram averages.

## 2 Alignment

### 2.1 Standard tilt-series alignment

**Fig. 1:**
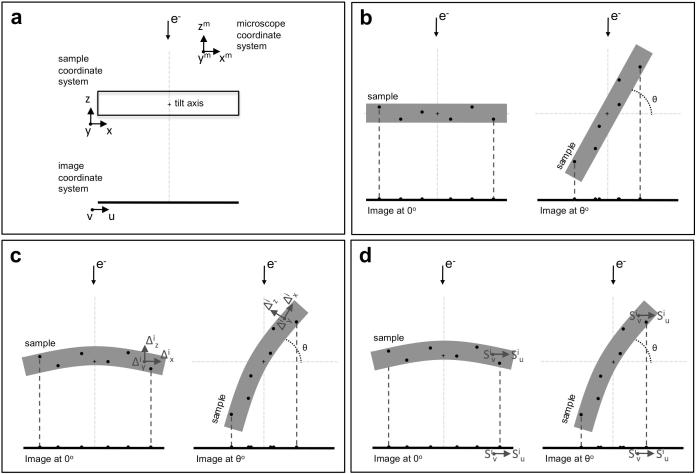
Introducing the sample deformation in tilt-series alignment. (a) The three different coordinate systems involved in the projection model: microscope, sample and projection image. The tilt axis is the Y-axis of the microscope. In this scheme, the tilt axis is marked by a crosshair in the middle of the sample and runs perpendicular to the sheet. At the untilted position, the coordinate system of the microscope and the sample coincide. The axes of the image system are denoted by *u* and *v*. In this simplified scheme, post-projection rotations are ignored, and *v* is thus parallel to the tilt axis, running perpendicular to the sheet. (b) Fiducial-based alignment relies on determination of the 3D coordinates of the fiducials (black spots in the sample) along with the basic image parameters by minimizing the differences between the calculated projections of the fiducials (black spots in the images) and the measured positions. Standard alignment ignores the potential deformation that the sample may undergo during imaging. (c) Sample deformation at the acquisition of any image *i* can be modelled by means of polynomial surfaces ($\Delta_{x}^{i}\text{,}\Delta_{y}^{i}\text{,}\Delta_{z}^{i}$) that account for the 3D motion at each point of the sample. The calculated projections of the fiducials are expected to better approach the experimental measurements. (d) Sample deformation at an image *i* can alternatively be modelled by polynomial surfaces ($S_{u}^{i}\text{,}S_{v}^{i}$) that represent the 2D motion at the projection image level for each point of the sample. This alternative modelling stems from the fact that the projection operation prevents full 3D description of the sample deformation with the approach in (c). See main text for details.

The most commonly used approach to tilt-series alignment in cryoET is based on the use of gold particles as fiducial markers (Lawrence, 1992, Mastronarde, 2006, Fernandez, 2012). The relationship between the 3D coordinates of the gold particles in the specimen and their coordinates in the images of the tilt-series is described by the projection model (Fig. 1a and b), given by the following system of equations (Lawrence, 1992, Mastronarde, 2006, Amat et al., 2010):

(1) $$p_{j}^{i}=M^{i}r_{j}+d^{i}\text{,}\;\;\;i=1\ldots N\text{,}\;\;\;j=1\ldots N_{m}$$

where

1. *N* is the number of images in the tilt-series.
2. $N_{m}$ is the number of fiducial markers.
3. $r_{j}=(x_{j}\text{,}y_{j}\text{,}z_{j})$ are the coordinates of the *j*-th fiducial marker in the specimen.
4. $M^{i}$ is the overall projection matrix and is given by $M^{i}=m^{i}{\mathrm{PR}}^{i}$, where
  (a) $R^{i}$ represents the 3×3 rotation matrix given by the Euler angles associated to the *i*-th image of the tilt-series. It includes the tilt angle ($\theta^{i}$) that rotates around the tilt axis, running along the Y-axis of the microscope coordinate system. It also includes the rotation angle ($\psi^{i}$) around the Z-axis of the microscope system (this rotation takes place after the projection operation, but can be included in this matrix as well). It might also include an initial tilt around the microscope X-axis.
  (b) P denotes the projection operation along the microscope Z-axis.
  (c) $m^{i}$ represents an isotropic scaling factor to account for magnification changes in the *i*-th image.
  Additional sophistication can be introduced in the projection model (e.g. anisotropic magnification, skewness) to represent more complicated specimen changes or technical imperfections during imaging (Luther et al., 1988, Diez et al., 2006, Lawrence et al., 2006, Mastronarde, 2006, Mastronarde, 2008).
5. $p_{j}^{i}=(u_{j}^{i}\text{,}v_{j}^{i})$ are the coordinates of the projection of the *j*-th fiducial marker in the *i*-th image.
6. $d^{i}$ represents the image shifts with respect to a reference center, normally placed at the centroid of the fiducials.

The parameters of this projection model (coordinates of the fiducials, $r_{j}$, and the parameters associated to the images, $m^{i}\text{,}\psi^{i}\text{,}\theta^{i}\text{,}d^{i}$) can be determined by solving an optimisation problem. It is commonly formulated as a non-linear least-squares problem aiming to minimize the following objective function:

(2) $$f=\overset{N}{\underset{i=1}{\sum }}\overset{N_{m}}{\underset{j=1}{\sum }}{\left\|q_{j}^{i}-p_{j}^{i}\right\|}^{2}=\overset{N}{\underset{i=1}{\sum }}\overset{N_{m}}{\underset{j=1}{\sum }}\left({\left(u_{j}^{i\prime }-u_{j}^{i}\right)}^{2}+{\left(v_{j}^{i\prime }-v_{j}^{i}\right)}^{2}\right)$$

which denotes the sum of squares of the residuals, i.e. distances between the measured ($q_{j}^{i}=(u_{j}^{i\prime }\text{,}v_{j}^{i\prime })$) and calculated ($p_{j}^{i}=(u_{j}^{i}\text{,}v_{j}^{i})$) positions of the fiducials. This function, though nonlinear, is easily differentiable, which allows optimisation by conjugate gradient or quasi-Newton methods (Press et al., 2002). The standard procedure for fiducial-based tilt-series alignment has been reviewed in detail elsewhere (Mastronarde, 2006, Amat et al., 2010).

### 2.2 Modelling the sample deformation as 3D motion

During imaging, the sample undergoes motion that can be largely described as doming motion (Brilot et al., 2012, Zheng et al., 2017). In cryoET, this motion results in the sample deviated from the single-tilt axis geometry during tilting. This is expected to have a detrimental effect in tilt-series alignment, and hence in the quality of tomograms.

In order to mitigate this problem, we have extended the tilt-series alignment approach by introducing sample deformation in the projection model (Eq. (1)), in which polynomial surfaces are used to represent the 3D motion of the fiducial markers in each image in the tilt-series:

(3) $$p_{j}^{i}=M^{i}(r_{j}+D_{j}^{i})+d^{i}\text{,}\;\;\;i=1\ldots N\text{,}\;\;\;j=1\ldots N_{m}$$

where $D_{j}^{i}=D^{i}(r_{j})=\left(\Delta_{x}^{i}(r_{j})\text{,}\Delta_{y}^{i}(r_{j})\text{,}\Delta_{z}^{i}(r_{j})\right)$.

The triad of polynomials $D^{i}(x\text{,}y\text{,}z)=\left(\Delta_{x}^{i}(x\text{,}y\text{,}z)\text{,}\Delta_{y}^{i}(x\text{,}y\text{,}z)\text{,}\Delta_{z}^{i}(x\text{,}y\text{,}z)\right)$ represents the 3D motion undergone by the sample in the *i*-th image by describing the shifts in the three directions, X, Y, Z, respectively, of the sample coordinate system (Fig. 1c). They are homogeneous polynomials of the form:

(4) $$P(x\text{,}y\text{,}z)=\overset{D_{z}}{\underset{l=0}{\sum }}\overset{D-l}{\underset{n=0}{\sum }}\overset{D-l-n}{\underset{m=0}{\sum }}P_{\mathrm{mnl}}\;x^{m}\;y^{n}\;z^{l}$$

with *D* and $D_{z}$ denoting the degree of the polynomial. If $D_{z}$ is set to 0, then bivariate polynomials are implemented (i.e. dependent only on X, Y and assuming there is no variation of the motion along the Z-axis of a relatively thin sample). Trivariate polynomials are expected to give a more precise description of the motion along Z, though at the expense of more parameters to determine. Note that the sample deformation in each image ($D^{i}$) is modelled independently from the other images to avoid any presumption about its evolution during the acquisition.

The number of coefficients of each of these polynomials is $(D_{z}+3)(D+2)(D+1)/6$. Quadratic (i.e. second degree) polynomial surfaces are adequate to geometrically approximate the sample doming (Zheng et al., 2017). Eq. (4) could then be expressed, for bivariate and trivariate polynomials respectively, as:

(5) $$\begin{array}{cc} P_{\mathrm{bivar}}(x\text{,}y\text{,}z) & =P_{00}+P_{10}\;x+P_{20}\;x^{2}+P_{01}\;y+P_{11}\;\mathrm{xy}+P_{02}\;y^{2}\\ P_{\mathrm{trivar}}(x\text{,}y\text{,}z) & =P_{000}+P_{100}\;x+P_{200}\;x^{2}+P_{010}\;y+P_{110}\;\mathrm{xy}+P_{020}\;y^{2}\;+P_{001}\;z+P_{101}\;\mathrm{xz}+P_{011}\;\mathrm{yz}+P_{002}\;z^{2} \end{array}$$

Therefore, we would need 18 (i.e. 3×6) or 30 (i.e. 3×10) parameters, for any image of the tilt-series, to model the 3D motion of the sample by using bivariate or trivariate quadratic polynomials, respectively. These parameters are determined by means of the same optimisation process that aims to minimize the sum of squared residuals (Eq. (2)).

### 2.3 Modelling the sample deformation as 2D motion at the image level

Since the image recorded on the detector is a 2D projection of 3D sample and the projection operation P is involved in the overall projection matrix $M^{i}=m^{i}{\mathrm{PR}}^{i}$ in Eq. (3), it may be difficult for the alignment process to obtain information of the motion along the electron beam direction (see section Results). Eq. (3) can be worked out to derive an equivalent formulation where the sample deformation is modelled by polynomial surfaces representing the 2D shifts of the fiducial markers at the projection image level (Fig. 1d):

(6) $$\begin{array}{cc} & p_{j}^{i}=M^{i}(r_{j}+D_{j}^{i})+d^{i}=M^{i}r_{j}+M^{i}D_{j}^{i}+d^{i}=M^{i}r_{j}+S_{j}^{i}+d^{i}\\ & i=1\ldots N\text{,}\;\;\;j=1\ldots N_{m} \end{array}$$

where $S_{j}^{i}=S^{i}(r_{j})=\left(S_{u}^{i}(r_{j})\text{,}S_{v}^{i}(r_{j})\right)$.

The set of polynomials $S^{i}(x\text{,}y\text{,}z)=(S_{u}^{i}(x\text{,}y\text{,}z)\text{,}S_{v}^{i}(x\text{,}y\text{,}z))$ represents the 2D motion perpendicular to the electron beam direction undergone by the sample in the *i*-th image, as seen at the image plane, with *u* and *v* denoting the two orthogonal directions of the image coordinate system (Fig. 1d). They are also homogeneous polynomials (Eq. (4)) and share all considerations expressed for the polynomials modelling the 3D sample motion $D^{i}(x\text{,}y\text{,}z)=\left(\Delta_{x}^{i}(x\text{,}y\text{,}z)\text{,}\Delta_{y}^{i}(x\text{,}y\text{,}z)\text{,}\Delta_{z}^{i}(x\text{,}y\text{,}z)\right)$ in Section 2.2. The relationship between these sets of polynomials is given by:

(7) $$S^{i}(x\text{,}y\text{,}z)=M^{i}D^{i}(x\text{,}y\text{,}z)$$

and the relationship between their coefficients can be expressed in matrix form as:

(8) $$\left(\begin{array}{c} S_{u\text{,}\mathrm{mnl}}^{i}\\ S_{v\text{,}\mathrm{mnl}}^{i} \end{array}\right)=M^{i}\left(\begin{array}{c} \Delta_{x\text{,}\mathrm{mnl}}^{i}\\ \Delta_{y\text{,}\mathrm{mnl}}^{i}\\ \Delta_{z\text{,}\mathrm{mnl}}^{i} \end{array}\right)$$

With this approach to tilt-series alignment, sample deformation at each image can be modelled by just two polynomials. Thus, the number of parameters required for any image would be reduced to 12 (i.e. 2×6) or 20 (i.e. 2×10) for bivariate or trivariate quadratic polynomials, respectively. Alternatively, it could also be possible to conduct the optimisation based on 3D motion (Section 2.2) followed by computation of the parameters for 2D motion according to Eq. (8).

### 2.4 Optimisation process and strategies to reduce the number of parameters

The total number of polynomial parameters involved in the alignment of a tilt-series of *N* images with the 2D motion approach is thus:

(9) $$N\times (D_{z}+3)(D+2)(D+1)/3$$

These parameters are to be added to those involved in the standard alignment procedure, namely coordinates of the fiducials and basic image parameters (at least image shifts) (see Section 2.1). Determination of in-plane rotations and tilts of the images is optional, as they could be determined precisely a priori, removing the need for its optimisation (Wan and Briggs, 2016). There is no need to determine image magnification either, as is inherently included in the polynomial model.

The inter-relationship among the alignment parameters may turn the optimisation into a difficult process, especially if the sample deformation is considered (Mastronarde, 2006, Wan and Briggs, 2016). We solved this issue by splitting the optimisation into two steps. The first step aims to set the reference tomogram by running the standard alignment. Thus, 3D coordinates of the fiducials and the image shifts are determined, and optionally the standard alignment parameters (rotation, magnification, tilt) are refined. In this step, the parameters from an external (e.g. IMOD) alignment can be adopted. The second step then intends to estimate the polynomial parameters that describe the motion undergone by the sample (represented by the reference tomogram). This is to account for the experimentally determined fiducial positions at the projection images that are deviated from the expected and motion-free positions.

The number of polynomial parameters to be fitted may prove to be a limiting factor for the practical applicability of our method. As any fiducial marker provides two measurements (i.e. its positions in *u* and *v*) on each image, the minimum number of fiducials required to determine the parameters is $(D_{z}+3)(D+2)(D+1)/6$. However, to increase the robustness of the alignment solution against imprecisions in the measured fiducial positions, it is desirable to have higher ratios of measurements versus parameters (Mastronarde, 2006, Amat et al., 2010).

In cases where the number of fiducials is limited, several options can be chosen to reduce the number of parameters. The polynomials can be forced to consist only of pure terms ($P_{m00}\text{,}P_{0n0}\text{,}P_{00l}$ in Eq. (4)) by removing the mixed terms. A less restrictive option would force pure terms only on Z ($P_{\mathrm{mn}0}\text{,}P_{00l}$). The polynomials resulting from those constraints are smoother and are expected to model the sample deformation less precisely. But they might still be useful in cases where the number of measurements is low. Other options to reduce the amount of parameters are the use of bivariate polynomials and lower degrees. In addition, constraining the polynomial coefficients on adjacently acquired images and enforcing smooth sample deformation through the tilt-series can further reduce the number of parameters (Mastronarde, 2006, Zheng et al., 2017).

### 2.5 Assessment of the modelling by cross-validation

The accuracy of the modelling depends on a number of factors such as the number of the fiducial markers, their distribution, the precision of their locations, the complexity of the true sample deformation, etc. These factors are shared by any other methods for landmark-based nonrigid image registration (Paul-Gilloteaux et al., 2017). While the modelling may properly describe the sample deformation in local areas covered by the fiducials used in the polynomial parameter fitting, it might misrepresent the deformation for other neighbour areas.

To estimate the quality of the modelling of the sample deformation, we have adopted the ‘leave-one-out’ (LOO) cross-validation test (Kukulski et al., 2011). Here, one fiducial marker is left out of the modelling and acts as an object of interest whose position is to be predicted. The fitting of the polynomials and the alignment are thus carried out with the remaining fiducials. Finally, the residual for the excluded fiducial, i.e. the distance between the actual, measured positions in the images of the tilt-series and the predicted positions according to the fitted model, is calculated. This process is repeated for all fiducial markers, and those residual values are averaged. This mean value, namely LOO residual, thus provides an assessment for the quality of the modelling of the sample deformation. A LOO residual lower than the mean residual obtained with the standard alignment indicates that the modelling is robust and valid for the area covered by the whole set of fiducials. Otherwise, it would suggest that the model might misrepresent certain areas, which would result in their reconstruction with poorer quality than the standard method.

## 3 Tomographic reconstruction

Along with the alignment procedure, we have implemented the associated reconstruction program that takes the sample motion into consideration. It is based on Weighted Backprojection (WBP). The program proceeds by reconstructing the slices perpendicular to the tilt axis, using simple backprojection. It makes use of the new projection model that incorporates either 3D motion (Eq. (3)) or 2D motion at the image projection level (Eq. (6)) to determine where the voxels of the tomogram (x) are projected:

(10) $$M^{i}\left(x+D^{i}(x)\right)+d^{i}=M^{i}(x)+S^{i}(x)+d^{i}$$

After backprojection, the reconstructed slices are weighted with ramp filtering, and optional apodisation, so as to yield the WBP reconstruction. The program may work with the original, unaligned tilt-series to avoid unnecessary intermediate interpolations.

The processing is well suited for parallel computing, and a multithreaded implementation is now available. Reconstruction of full-sized tomograms (e.g. 4000×4000×1000 from 40 images) requires around 1-2 h in standard desktop computers equipped with 4–8 cores. The approach based on 2D motion is around 20% faster than that on 3D motion since the 2D motion is independent of the projection operation (compare both sides of Eq. (10)). Therefore, there are less dependences in the program and precomputation of values, in particular related to projection of voxels, becomes possible.

As an additional function, the program is also able to track any defined position in the tomogram through the tilt-series while taking the sample motion into account. This allows extraction of subtiltseries associated to the subtomograms, where the sample motion is already compensated. Reconstruction of subtomograms directly from their subtiltseries is therefore possible by standard WBP using standard tomographic programs (e.g. IMOD (Kremer et al., 1996) or tomo3d (Agulleiro and Fernandez, 2011, Agulleiro and Fernandez, 2015)) or by Fourier inversion using, for instance, RELION (Scheres, 2012, Scheres, 2012).

## 4 Results

### 4.1 Test datasets and methods

We tested our methods on three datasets. The first two datasets (ribosome and proteasome) are representative of relatively thin samples (∼25 nm and ∼15 nm, respectively). The third sample, the basal body, is an example of a thick specimen (∼300 nm). Quantifoil R 2/2, 200 mesh, Copper/Rhodium, holey carbon grids were used for all samples. Table 1 summarizes the acquisition details and the obtained results.

**Table 1:** Test datasets. Details and summary of results.

	Ribosome	Proteasome	Basal body
*Acquisition details*			

N. tilt-series:	7	14	6
Tilt range, interval:	±60°,3°	±60°,3°	±60°,1°
N. images/tilt-series:	41	42	121
N. frames/image:	3	5	5
Accumulated dose:	$60\;{\text{e}}^{-}/{Å}^{2}$	$60\;{\text{e}}^{-}/{Å}^{2}$	$60\;{\text{e}}^{-}/{Å}^{2}$
Defocus:	3–5 μm	1–4 μm	1–4 μm
Pixel size:	2.17 Å	2.56 Å	4.82 Å
N. fiducials:	6–11	26–55	22–43
N. subtomograms:	3120	3928	1980
			
*Results of alignment*			
Avg. mean residual (standard alignment)	1.95	1.83	1.68
Avg. mean residual (bivariate motion)	0.75	0.78	1.36
Avg. mean residual (trivariate motion)	0.62	0.63	0.99
Avg. ratio measures/unknowns (bivariate motion)	1.42	7.39	5.88
Avg. ratio measures/unknowns (trivariate motion)	1.15	4.43	3.53
Avg. LOO residual (bivariate motion)	4.34	0.92	1.66
Avg. LOO residual (trivariate motion)	4.18	1.00	1.50
			
*Results of subtomogram averaging*			
Resolution (standard alignment):	13.8 Å	12.0 Å	30.4 Å
Resolution (new alignment):	12.6 Å	9.0 Å	29.0 Å

The first dataset was comprised of 7 tilt-series of purified 80S ribosomes from *Saccharomyces cerevisiae* (Bharat and Scheres, 2016). They were taken on a FEI Titan Krios microscope equipped with a K2 camera operated in counting mode, and with a pixel size of 2.17 Å. Data were acquired in the tilt range ±60° at 3° interval, in two branches starting at 30°, at 3–5 μm underfocus with a cumulative dose of $60\;{\text{e}}^{-}/{Å}^{2}$. Each tilt image was dose-fractionated into three image frames that were aligned with the Digital Micrograph software. A number of fiducials of 6–11 were available for tilt-series alignment. A total of 3120 subtomograms were extracted from the tomograms. The same dataset was used in (Bharat and Scheres, 2016) and is deposited at the EMPIAR database (Iudin et al., 2016) under accession number EMPIAR-10045. Further details about the imaging process are available in (Bharat et al., 2015, Bharat and Scheres, 2016).

The second dataset consisted of 14 tilt-series of purified 20S proteasomes from *Thermoplasma acidophilum*. They were taken on a FEI TF30 Polara microscope equipped with a GIF (Gatan energy filter) and a K2 camera operated in counting mode, using a pixel size of 2.56 Å. Data were acquired in the tilt range ±60° at 3° interval, in two branches starting at -21°, at 1–4 μm underfocus with a cumulative dose of $60\;{\text{e}}^{-}/{Å}^{2}$. Each tilt image was dose-fractionated into five image frames that were aligned with MotionCor (Li et al., 2013). Tilt-series had 26–55 fiducials for the alignment. A total of 3928 subtomograms were extracted from the tomograms.

The third dataset was composed of 6 tiltseries of purified basal bodies (BBs) from *Chlamydomonas reinhardtii* (Li et al., 2012). They were collected on the same FEI TF30 Polara microscope and essentially under the same imaging and cumulative dose conditions and same frame-alignment protocol as the 20S proteasome dataset. Here, the tilt-series consisted of 121 images in the tilt range ±60° at 1° increment that were acquired in two branches starting at 0° with a pixel size of 4.82 Å. A number of fiducials of 22–43 were available for tilt-series alignment. A total of 1980 subtomograms (BB triplets) were extracted from 10 BBs found in the tomograms.

Standard alignment (including rotation, magnification, tilt) and tomographic reconstruction with WBP of all tilt-series was performed with IMOD (Kremer et al., 1996) and tomo3d (Agulleiro and Fernandez, 2011, Agulleiro and Fernandez, 2015), respectively. Alignment with consideration of the motion was carried out by using the IMOD standard alignment as the first step, followed by the polynomial modelling of the motion as described above. Reconstruction of tomograms with motion-compensation then followed. Positions of subtomograms were obtained from the standard tomograms, automatically with template matching using MolMatch (Forster et al., 2010) or Spider (Frank et al., 1996), or by manual picking. Subtomogram averaging of the ribosome dataset was done with RELION (Scheres, 2012, Scheres, 2012), with the methods and protocols described in (Bharat et al., 2015, Bharat and Scheres, 2016). For the proteasome and BB datasets, CTF correction was applied with TomoCTF (Fernandez et al., 2006) and subtomogram averaging was carried out with MLTOMO (Scheres et al., 2009), using D7 symmetry with the proteasome. In all cases, the gold standard procedure was used to conduct subtomogram averaging (Scheres and Chen, 2012, Chen et al., 2013). Resolution was assessed against a high-resolution single particle cryoEM map of the same specimen in the ribosome and proteasome cases.

### 4.2 Alignment based on 3D motion and on 2D motion

We applied the 3D motion-based alignment (Section 2.2) to all tilt-series and observed a decrease of the residual with regard to the standard alignment. This residual reduction is essentially the same as that obtained with the 2D motion-based approach (Section 2.3), which will be reported in the following section.

**Fig. 2:**
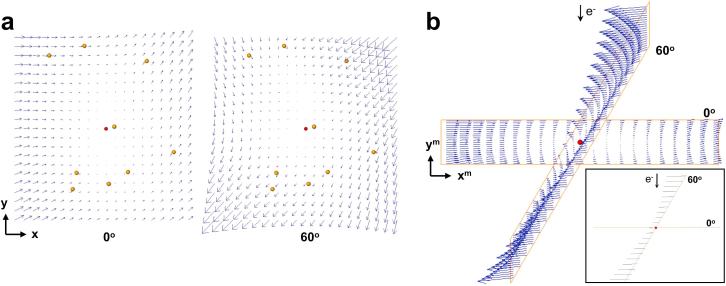
Beam-induced motion estimated by the 3D motion-based alignment process. One representative ribosome tilt-series is used as an example. (a) Local 3D shifts $\left(\Delta_{x}^{i}\text{,}\Delta_{y}^{i}\text{,}\Delta_{z}^{i}\right)$ given by the polynomial surfaces (bivariate, second degree) that were fitted for the images at 0° (left) and 60° (right). Only the XY components of the vectors are clearly observable in this view (the Z component runs perpendicular to the sheet). Fitted 3D fiducial coordinates are represented with yellow dots. The red dot represents the centroid of the fiducials. The panels show the motion for a field of view of 1 $\mu {\text{m}}^{2}$ around that centroid. Vectors are magnified by 20×. X- and Y- axes of the sample coordinate system are indicated. (b) Beam-induced motion patterns shown in (a) are presented in the microscope coordinate system, i.e. according to the tilt angle of the views. Note that all the vectors run perpendicular to the electron beam. This is better observed in the inset, which is viewed from the tilt axis and arrowheads have been removed. X- and Y- axes of the microscope coordinate system are indicated and the Z-axis runs along the electron beam direction.

To monitor the alignment and analyse the sample deformation, we visualized the vectors representing the local 3D motion as estimated by the alignment process and we saw that they were perpendicular to the electron beam direction. For illustrative purposes, Fig. 2(a) shows the local 3D motion obtained for one of the ribosome tilt-series. The alignment was carried out with bivariate quadratic polynomials. The local 3D shifts for the images at 0° (left) and 60° (right) are presented. The interesting point arises when these shifts are visualized in 3D and placed in the microscope coordinate system, that is, according to the corresponding tilt angle (Fig. 2(b)). This view shows that the optimisation process manages to estimate only the components of the motion perpendicular to the electron beam, but not the motion along the beam direction. Regardless of the tilt angle, all vectors run parallel to the XY-plane of the microscope system.

These results indicated that the 3D motion-based approach only manages to determine projections of the true 3D motion of the sample. They led us to derive an equivalent approach by introducing the sample deformation in the alignment directly based on 2D motion at the projection image level, as described in Section 2.3. The results presented in the following sections were obtained with this approach.

### 4.3 Reduction of the residual with motion-aware alignment

**Fig. 3:**
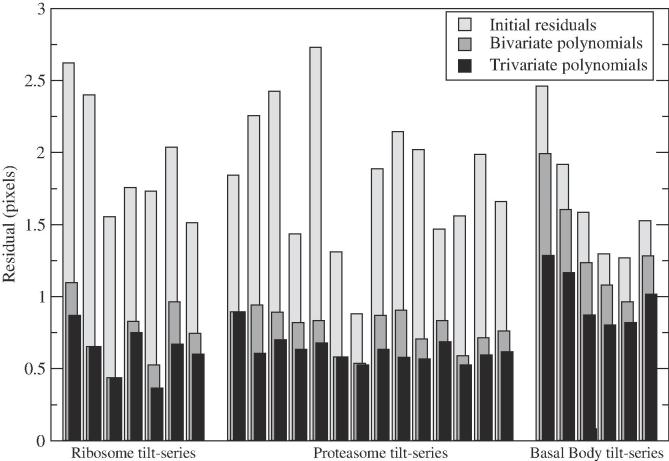
Mean residual for all datasets.

We applied our alignment procedure to all datasets using both bivariate and trivariate quadratic polynomials. Fig. 3 illustrates the mean residual for each individual tilt-series tested in this work. The standard alignment results in a residual mostly in the range [1.25,2.5] pixels, with average values of 1.95, 1.83 and 1.68 (ribosome, proteasome and BB, respectively). Motion compensation with either type of polynomials improves the results. For thin samples (ribosome and proteasome), the residual decreases and ranges mainly in [0.5,1.0] with average values around 0.76 (bivariate) and 0.63 (trivariate). In general, for these thin samples there is little difference between bivariate and trivariate polynomials. By contrast, for the thick sample (BB) only trivariate polynomials enable an average residual below 1.00 pixels and with all tilt-series in the range [0.75,1.25]. These results point out that there may exist an important variation of the motion from top to bottom of thick samples, which only these trivariate polynomials may account for.

**Fig. 4:**
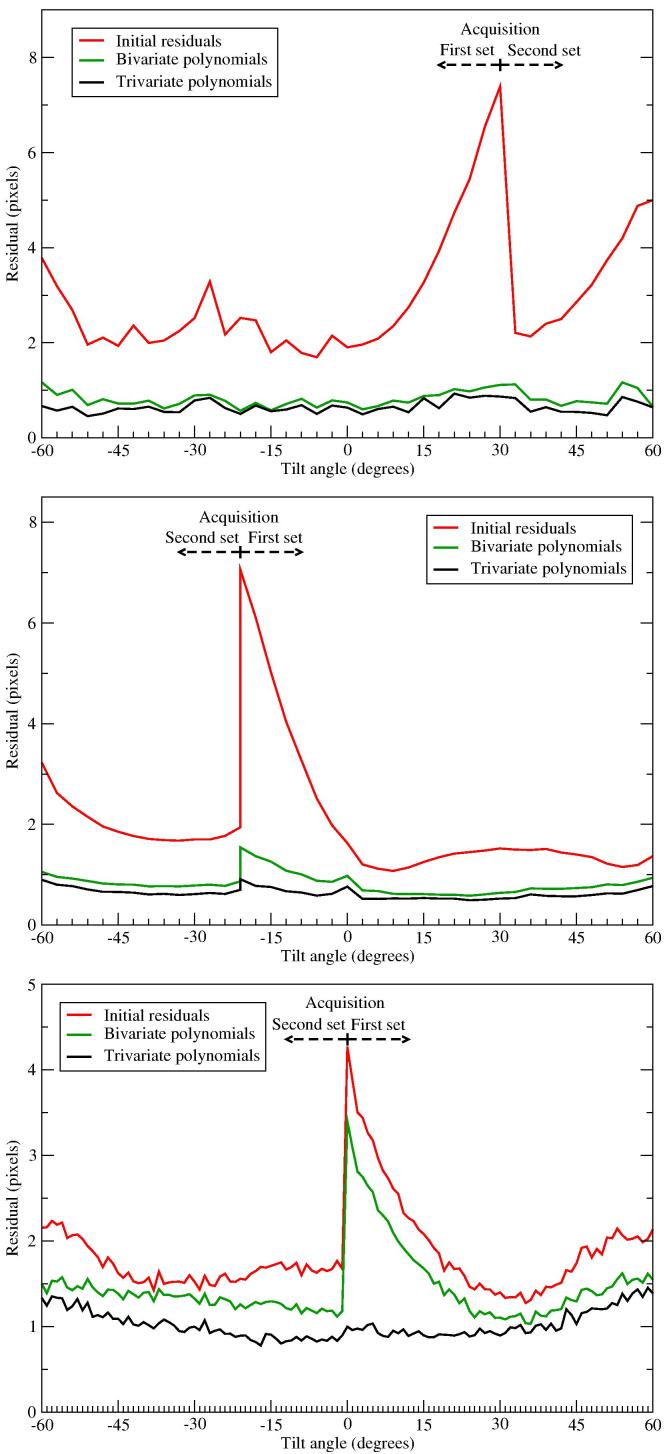
Averaged alignment residual as a function of the acquired image. Averaged residual calculated from 2300, 17645 and 25624 individual residuals collected over the tilt-series from the ribosome (top), proteasome (middle) and Basal Body (bottom) datasets. Tilt-series acquisition was done in two branches that are marked in the plots. The green and black curves present the residuals from the alignment considering motion, using bivariate and trivariate polynomials, and the red one corresponds to the standard alignment. Here the original tilt angles from the goniometer were preserved.

To analyse the evolution of the residual with the acquisition process, we collected individual residuals (i.e. $\left\|q_{j}^{i}-p_{j}^{i}\right\|$ in Eq. (2)) and averaged them as a function of the acquired imaged, as shown in Fig. 4. The red curves show that the residual from the standard alignment is always the highest at the beginning of the acquisition and progressively reduces and reaches a plateau. This behaviour in reduction of the residual is true for all the datasets we have tested. It reveals the relative magnitude of the sample motion throughout the tilt-series. This suggests that the beam-induced motion is highest at the beginning of the acquisition, around the first 8 or 24 images for the ribosome, proteasome or BB datasets, respectively, which correspond to an accumulated exposure of around 12 ${\text{e}}^{-}/{Å}^{2}$.

The new alignment substantially reduces the residual for all images, in particular the first images, as shown in Fig. 4. The comparison between bivariate and trivariate polynomials (green and black curves, respectively) strengthens the above observation about their performance with the sample thickness.

Table 1 presents the overall residual results, averaged from all tilt-series, for all datasets. The table also includes the overall LOO residual and ratio measurements/unknowns. The very few fiducials in the ribosome tilt-series (in the range 6–11) make this ratio only slightly higher than 1. This may imply a modelling of the sample deformation with limited quality, as confirmed by the LOO residual (greater than 4) higher than the residual from the standard alignment (1.95 pixels). As a consequence, there is risk that some areas of the motion-compensated tomograms might be poorer than in the original ones. In the proteasome and BB cases, however, there were plenty of fiducials (in the range 22–55) and the ratio measurements/unknowns proves to be high (3.53–7.39). Moreover, their LOO residual is better than that of the standard alignment, thereby indicating a good modelling of the sample deformation by the corresponding polynomial functions. The LOO residual of the BB dataset also reinforces the importance of trivariate polynomials for this thick sample (1.50 pixels against 1.66 from bivariate ones). For the proteasome, bivariate polynomials obtain slightly better LOO residual (0.92 vs. 1.00) because the limited Z-distribution of fiducials in this thin sample prevents optimal modelling of the motion variation across its thickness.

### 4.4 Improvement in tomographic reconstruction

**Fig. 5:**
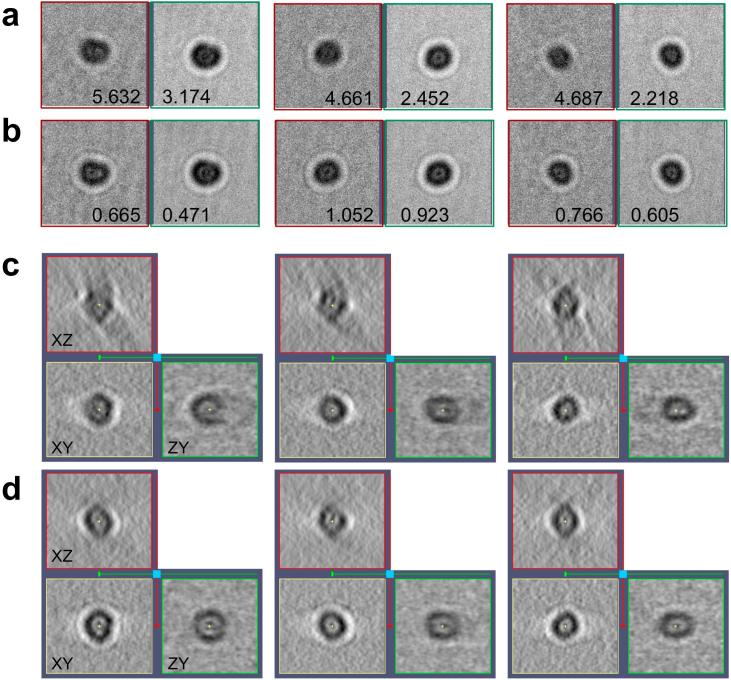
Effect of motion-compensated tomographic reconstruction on gold particles. (a and b) Subtiltseries associated to three gold particles with the standard alignment (a) and with motion considered (b). The panels show the average of the 10 first acquired images (left panels, in red) and the remaining 31 images of the subtiltseries (right panels, in green). The residual (in pixels) associated to those two parts of the subtiltseries is indicated. The higher the residual is, the more blurred the average in the panel appears. (c,d) Reconstruction of the gold particles resulting from the standard (c) and new (d) alignment. Central planes XY, XZ and ZY are shown.

After alignment with motion taken into account, we used our new program to reconstruct the tomograms. The improvement in tomogram quality is evident in all three cases. The effects are especially striking around the gold particles serving as fiducial markers. Compared to the standard results, there are fewer artefacts in their surroundings. Fig. 5 presents three gold particles from a representative ribosome tilt-series that exhibited high residual (3.77, 2.99 and 2.83 pixels) with the standard alignment. To illustrate the effect of the alignment, subtiltseries associated to them are displayed in Fig. 5(a), highlighting the uneven distribution of the residual, much higher at the first acquired images (hence blurred averages in the corresponding panels). The new alignment scheme reduced the residual (0.518, 0.955, 0.645 pixels) and balanced its distribution, showing clearer averages in the panels of Fig. 5(b). The improvement is particularly evident at the first acquired images (compare the left panels of the subtiltseries in Fig. 5(a and b)). The misalignment in Fig. 5(a) causes artefacts around the reconstructed particles (Fig. 5(c)), which are absent in the new reconstruction (Fig. 5(d)).

**Fig. 6:**
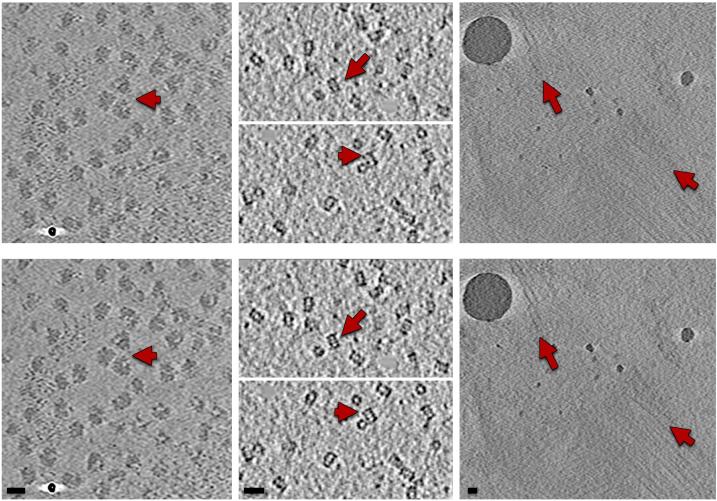
Effect of motion-compensated tomographic reconstruction on biological features in tomograms. Areas of tomograms from datasets of (from left to right) ribosomes, proteasomes and BBs reconstructed from the standard (top) and new (bottom) alignment. Arrows point to areas where the improvements are particularly remarkable. Scale bars: 20 nm.

Though subtler, the improvement in tomogram quality can also be observed in biological features, as Fig. 6 illustrates with selected tomograms from the three datasets. In all examples, the reconstruction looks less noisy, cleaner and sharper. The improvement in some ribosomes and proteasomes is outstanding, so is the enhancement of some microtubule walls in the BBs. In these tilt-series, the standard alignment resulted into a mean residual of 2.62, 2.73 and 2.46 whereas the new alignment reduced to 1.097, 0.83 and 1.29 pixels, respectively.

### 4.5 Improvement in subtomogram averaging

We further evaluated our new method by subtomogram averaging. For this purpose, we chose the tilt-series alignment parameters that resulted in the lowest LOO residual. Thus, we used trivariate polynomials for the thick BB sample and bivariate ones for the proteasome dataset (Table 1). For the ribosome dataset, we further tuned the alignment parameters to improve the LOO residual (3.26) and the ratio measures/unknowns (around 2.0). This was achieved by using bivariate polynomials and reducing the order in some tilt-series.

**Fig. 7:**
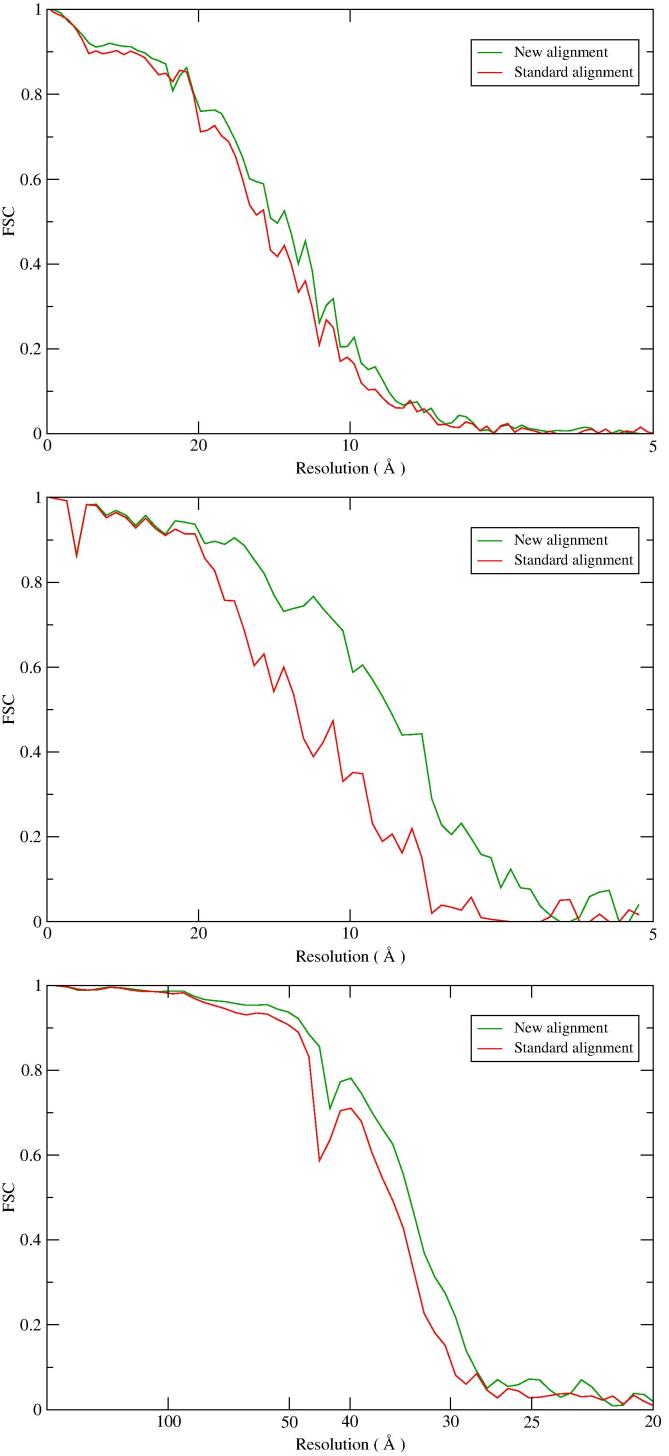
**FSC curves from subtomogram averaging.** Ribosome (top), proteasome (middle) and Basal Body (bottom). The FSC curves for the ribosome and proteasome were computed from the subtomogram average from all particles against a high-resolution cryoEM map. For the BB, the FSC curves come from the gold standard procedure, and were computed from random halves of the data.

Subtomograms were extracted from the ribosome, proteasome and BB tomograms and subjected to subtomogram averaging with the methods mentioned in Section 4.1. Fig. 7 presents the Fourier Shell Correlation (FSC) curves and confirms that the alignment with consideration of the motion translates into better subtomogram averages, with improved resolution. The curves for the ribosome and proteasome were computed against high-resolution single particle cryoEM maps (Bai et al., 2013, Grant and Grigorieff, 2015) while for the BB the Gold-Standard FSC curves from the two half-data are shown.

We saw improvement in resolution in all three cases. Estimated at FSC 0.5 (ribosome and proteasome) and 0.143 (BB), the resolution gains are in the range 1–3 Å, as summarized in Table 1. Specifically, the resolution of the three averages increased from 13.8 to 12.6 (ribosome), from 12.0 to 9.0 (proteasome), and from 30.4 to 29.0 Å (BB triplet). Although the improvement in resolution is modest, the FSC curves obtained from the new alignment are all raised higher than from the standard alignment (Fig. 7), demonstrating the robustness of the new method. Supplementary Fig. S1 shows the effect of the new alignment in the subtomogram averaging density maps. In the proteasome case in particular, the improved resolution allows some secondary structure elements to become discernible.

Finally, in order to analyze the motion in more detail, the sample motion was evaluated at the frame level. We focused on the proteasome datasets as the gold particles could be readily discernible in all the frames. We applied the standard and the bivariate polynomial motion-aware alignment to each supertilt-series consisting of a total of 210 acquired frames (42 tilts, 5 frames per tilt). The evolution of the residual from the standard alignment with the acquisition process (Supplementary Fig. S2) exhibited the same general trend as that observed with the standard tilt-series (Fig. 4), i.e. largest in the very first frames and reaching a steady state after around 12 ${\text{e}}^{-}/{Å}^{2}$. The residuals within each tilt suggest motion caused by the mini-exposure series at each tilt, with the largest movement at the first frame followed by gradual relaxation in the following 4 frames. The relative difference in residual among the 5 frames at each tilt is much smaller compared to the absolute scale in the entire tilt-series. After tomographic reconstruction and subtomogram averaging, we observed no improvement in resolution, and the FSC curve (Supplementary Fig. S3) was very similar to that presented in Fig. 7. Thus, the new alignment at the frame level provides no advantage over the standard whole-frame alignment applied individually at each tilt, at least at the current resolution.

## 5 Discussion and conclusions

In this work, we have extended the standard fiducial-based alignment procedure in cryoET by introducing the sample motion in the projection model. The doming of the sample during tilt-series acquisition is modelled by quadratic polynomials that represent the motion in different directions. The new scheme improves tilt-series alignment according to the reduced mean residual. A companion 3D reconstruction program makes use of the motion estimated in the alignment to obtain the motion-compensated tomograms with increased quality and higher level of detail. The improvement is also demonstrated by subtomogram averages with better FSC curves, thereby indicating that the new procedure is robust and has potential to be useful in cryoET to increase the resolution.

In the course of this work we observed that our initial formulation, where the sample deformation was modelled with 3D motion at the sample level, only managed to estimate the components of the motion that are perpendicular to the electron beam direction. This led us to derive an equivalent formulation directly based upon 2D projection of this 3D motion onto the image plane. It yields identical alignment results, but requires fewer parameters. We also observed substantial variation of the motion across the sample thickness in the case of thick specimens, as modelled by trivariate polynomials. However, it is rather subtle for thin samples, suggesting that bivariate polynomials may be adequate for their motion modelling.

The evolution of the residual from the standard alignment suggests that the beam-induced sample motion is largest at the beginning of tilt-series acquisition and progressively slows down, consistent with previous observations (Brilot et al., 2012, Li et al., 2013). This enables a quantitative understanding of the beam-induced motion during data collection in cryoET. Another interesting point is that the per-frame electron dose employed here is in the range 0.1–0.5 ${\text{e}}^{-}/{Å}^{2}$, about 3–6-fold less than that typically used in single particle cryoEM. Even with such low dose, the sample undergoes considerable motion, as evident by the large alignment residual in the first acquired images (frames). This is in agreement with previous observations in the field (Typke et al., 2007, Brilot et al., 2012, Zheng et al., 2017). Our results suggest that, at each tilt, the sample undergoes the largest motion with the renewed exposure at the first frame and then relaxes, as observed by Brilot et al. (2012).

One of the limitations of our motion-aware alignment procedure is the dependence on fiducials. Their scarcity or uneven distribution could potentially result in over-fitted solutions where the estimated motion is accurate enough locally around the fiducials, but not reliable for the entire field of view. As a consequence, there may be areas of the resulting tomogram with deteriorated quality compared to the standard approach. To evaluate the reliability of the alignment and detect these potential circumstances, we have adopted the cross-validation strategy from Kukulski et al. (2011) to provide the LOO residual as a complementary metric. It reinforces the information given by the ratio measurements/unknowns to detect scarcity of fiducials and complements it to identify their limited spatial distribution. Another presumption is that the movement of the fiducials is a faithful reflection of the movement of the biological material. This needs to be further tested in the future. However, our procedure could potentially be applicable with biological features serving as virtual fiducials, provided that there is enough contrast in the images to track them accurately, as typically used in fiducial-less alignment methods (Brandt, 2006, Sorzano et al., 2009, Castano-Diez et al., 2010, Amat et al., 2010, Han et al., 2014).

The number of parameters of our procedure might limit its applicability. Several strategies described in Section 2.4 can help reduce the requirements. As mentioned above, the residual curves from the standard alignment suggest that the changes in the sample occur gradually during data acquisition. This supports application of temporal constraints to the parameters to enforce smoothly time-varying sample motion, similarly to IMOD or MotionCor2 (Mastronarde, 2006, Zheng et al., 2017). This may result in a substantial reduction of the parameters and improvement of the robustness of the modelling.

Despite the significant improvement of the alignment residual with our test datasets, the resolution of the subtomogram averages still falls short of expectations. Several factors may be involved, including the limited number of subtomograms in our three tests, and also their relatively larger pixel size compared to state-of-the-art single particle cryoEM. We believe that the limited amount of fiducials in the ribosome dataset prevents proper modelling of the underlying sample deformation owing to over-fitting problems. In the proteasome and BB datasets, we speculate that a major limiting factor is CTF determination and correction, carried out here by a 2D procedure (Fernandez et al., 2006). Emerging strategies for CTF estimation and correction in 3D (Bharat et al., 2015, Galaz-Montoya et al., 2016, Kunz and Frangakis, 2017, Turonova et al., 2017) are expected to improve the resolution.

The software package, tomoalign, that implements the alignment and tomographic reconstruction presented here will be available for public use through our web site. The package has been made compatible with IMOD to facilitate integration in the standard workflow used in cryoET.
