# Supplementary material for: Cryo-tomography tilt-series alignment with consideration of the beam-induced sample motion
Source: J Struct Biol. 2018 Jun;202(3):200–9. doi: 10.1016/j.jsb.2018.02.001 (PMC5949096; doi:10.1016/j.jsb.2018.02.001)
Supplement: Supplementary data [file mmc1.pdf]

Supplementary Figures

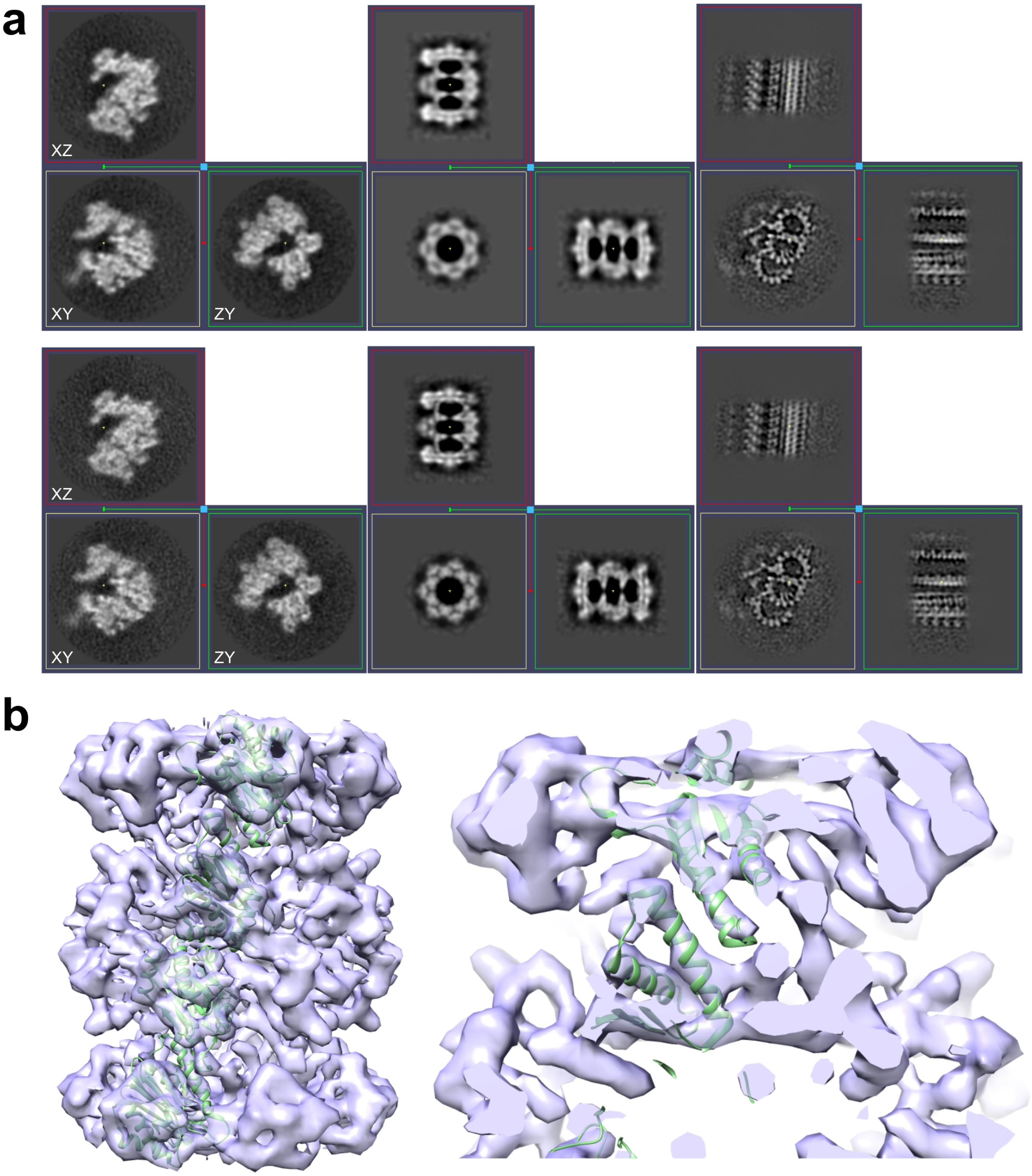

Figure S1: **Subtomogram average maps.** (a) Selected XY, XZ and ZY planes from the density maps of the ribosome, proteasome and basal body triplet (from left to right), obtained with the standard alignment (top) and the motion-aware method (bottom). The small yellow crosshairs indicate the correspondence between the XY, XZ and ZY planes of the 3D maps. (b) Sharpened subtomogram average map of 20S proteasome at 9 Å obtained from tomograms calculated with the new alignment method. Consistent with the estimated resolution, discernable rod-shaped structures fit well with the atomic structure (pdb 3j9i).

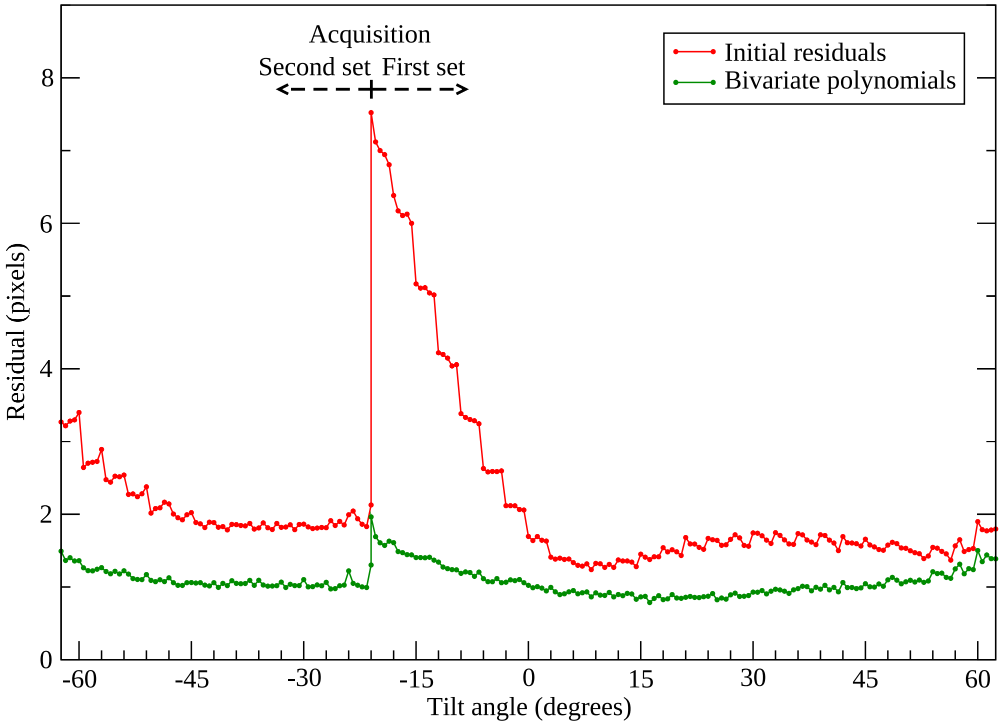

Figure S2: **Averaged alignment residual as a function of the acquired frame.** Averaged residual calculated from 88225 individual residuals collected over the super-tilt-series from the proteasome datasets. Tilt-series acquisition was done in two branches that are marked in the plots. For each tilt, five points are presented representing the 5 frames per tilt. The acquisition order of the frames follows that marked in the plot (first set, from left to right; second set from right to left). The red curve present the average residual from the standard alignment while the green curve corresponds to the bivariate polynomial motion-aware approach.

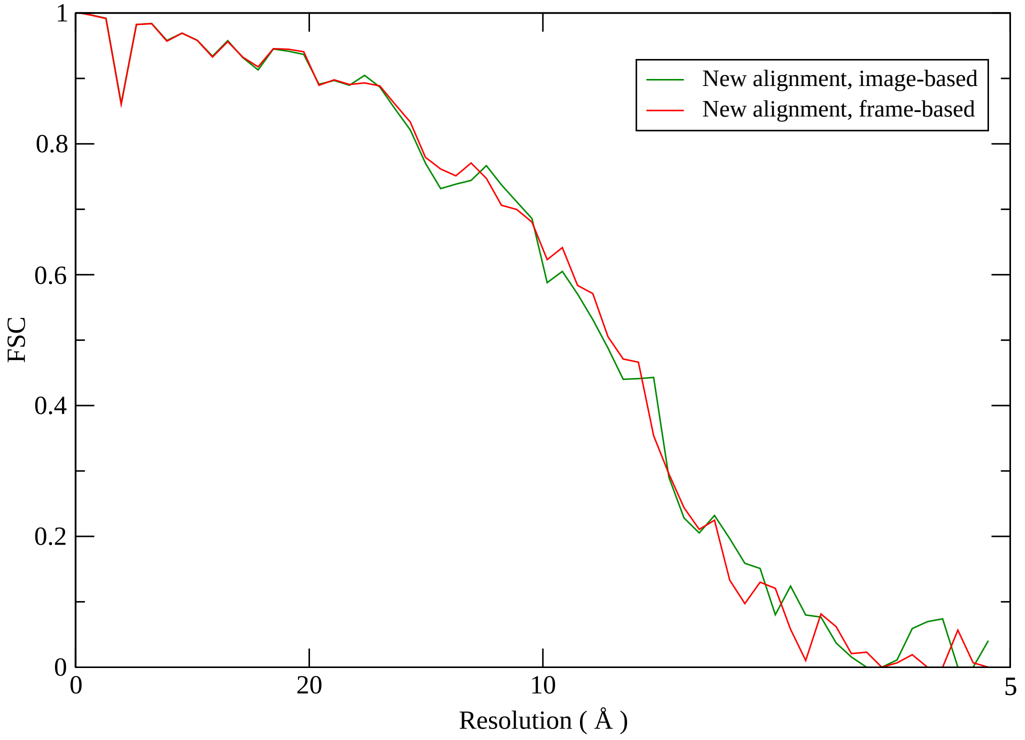

Figure S3: **FSC curves from subtomogram averaging using the new tilt-series alignment applied at the frame level and at the image level.**
